# Enhanced Arithmetic Optimization Algorithm for Parameter Estimation of PID Controller

**DOI:** 10.1007/s13369-022-07136-2

**Published:** 2022-08-26

**Authors:** Mohamed Issa

**Affiliations:** 1 Computer and Systems Department, Faculty of Engineering, Zagazig University, Zagazig, Egypt; 2 Faculty of Computer Science, NAHDA University in Beni-Suef, New Beni Suef City, Egypt

**Keywords:** PID controller, Arithmetic optimization algorithm (AOA), Harris Hawk optimization algorithm (HHO)

## Abstract

The Proportional-Integral-Derivative (PID) controller is a key component in most engineering applications. The main disadvantage of PID is the selection of the best values for its parameters using traditional methods that do not achieve the best response. In this work, the recently released empirical identification algorithm that is the Arithmetic Optimization Algorithm (AOA) was used to determine the best values of the PID parameters. AOA was selected due to its effective exploration ability. Unfortunately, AOA cannot achieve the best parameter values due to its poor exploitation of search space. Hence, the performance of the AOA exploit is improved by combining it with the Harris Hawk Optimization (HHO) algorithm which has an efficient exploit mechanism. In addition, avoidance of trapping in the local lower bounds of AOA–HHO is enhanced by the inclusion of perturbation and mutation factors. The proposed AOA–HHO algorithm is tested when choosing the best values for PID parameters to control two engineering applications namely DC motor regulation and three fluid level sequential tank systems. AOA–HHO has superiority over AOA and comparative algorithms.

## Introduction

PID controller is used in manufacturing industries for process control due to their effectiveness, robustness and durability [1]. PID controller has common control parameters such as system stability, the time it takes for the process to settle (settling time), and bypass and error between the desired response and the actual response [1]. Due to the sharing of processes in factories, parameter setting is an important task, and proper configuration allows to obtain efficient transient performance in terms of minimum settling time, steady-state error, maximum bypass and rise time as possible. The three parameters of PID controller are proportional gain (K_p_), integral gain (K_i_) and derivative gain (K_d_).

The main advantages of PID controller are concluded as in the following [1]:

1. The P controller is used for stabilizing the gain and producing a constant steady-state error
2. The I controller is used to eliminates or decreases the steady-state error
3. The D controller is used to decrease the rate of change of error, overshoot and settling time.

PID controller is used to regulate many industrial process such as pressure, temperature, flow rate, feed rate, weight, speed and position [1]. There are three categories for tuning the parameters of the PID controller, which are analytical methods, rule-based methods, and numerical methods [2]. The most common method is Ziegler−Nichol (ZN) [3] to be the classic method for adjusting parameters of the PID controller and has been classified as analytical method. ZN does not offer the best performance.

Heuristic optimization algorithms can be used to tune PID's parameters that have been classified as numerical methods and have been popular in the literature. Stochastic optimization methods such as heuristic algorithms [4] was suitable for tuning PID parameters because it treats the problem as a black box and adjusts the parameters and fitness tracking (fitness function) to achieve the optimum value. A meta-heuristic algorithm is a search-based algorithm that speeds up the exploration of problem's search space depending on a random motion to detect an acceptable solution in an acceptable time [4]. The meta-heuristic algorithm mimics the search methods from physics, humans, or nature. Sine–Cosine Optimization algorithm (SCA) [5] that drills into the search space by drawing search agents toward the best-established region based on cosine and sine factors. Besides, the Particle Swarm Optimization algorithm (PSO) [6] simulates the search strategy of birds flowing from nature. Also, there are a lot of released algorithms such as Ions Motion Optimization (IMO) [7], Lightning Attachment Procedure Optimization [8], Moth-Flame Optimization (MFO) [9], and other hundreds of algorithms are developed.

Meta-heuristic algorithms have successfully improved many engineering problems in fields as diverse as bioinformatics [10–17], Motor design [18, 19], Solar Energy [20], Robot design [21], Passive suspension system optimization [22] and many others. Many options are available to design the controllers with a lot of meta-heuristics algorithms in the literature. Such as particle swarm optimization algorithm was used for finding the optimum design of PID controller in the AVR systems [23] [24].

**Fig. 1:**
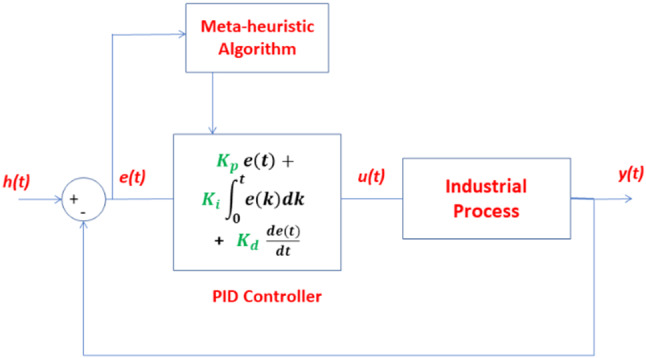
Adjust PID parameters through meta-heuristic algorithm

As shown in Fig. 1, meta-heuristic algorithm adjust the three parameters of the PID controller to improve the process response performance where e(t) and u(t) are the input and output signals of the PID controller, respectively. e(t) is the error signal which is the difference between the set point signal (h(t)) and the output response of the process to be controlled (y(t)). u(t) represents the controlled signal that output from the PID controller and applied on the process to be controlled.

The objective function used to improve process performance is the minimum integral to absolute error (IAE). It is the sum of the differences between the desired response (h(t)) and the actual response (y(t)) which is represented as in Eq. (1) [1].

1 $$IAE= {\int }_{0}^{\infty }\left|y\left(t\right)-h(t)\right| \mathrm{ dt}$$

**Fig. 2:**
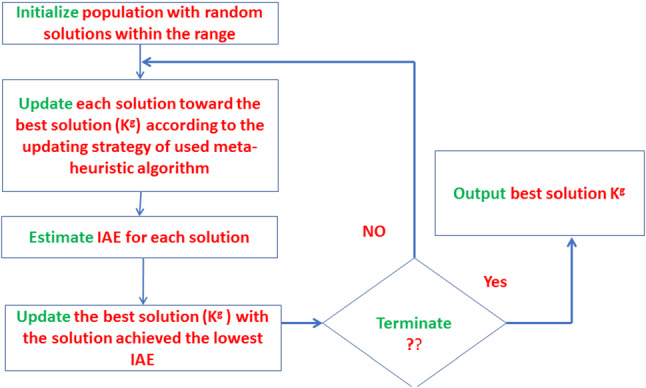
The flowchart of tuning the PID’s parameters using meta-heuristic algorithms

The procedure of estimating the values of PID’s parameters (K_p_, K_i_ and K_d_) as shown in Fig. 2. The solutions are updated using the updating strategy of the meta-heuristic techniques for several iterations. During each iteration, the best solution K^g^ (K^g^_p_, K^g^_i_ and K^g^_d_) which produce the best IAE is updated with the best solution founded during updating of solutions.

In the literature, there are several improvements in meta-inference for optimal design of a PID controller such as a constraint PSO (CPSO) [25], dynamic PSO (dPSO) [26], opposition-based Henry gas solubility optimization algorithm (OBL-HGS) [27], and improved whale optimization algorithm (IWOA) [28]. Besides various other descriptive algorithms that have been used to optimize the parameters of the PID controller to improve the performance of DC motor as Invasive Weed Optimization (IWO) [29], Flower Pollination Algorithm (FPA) [30], FireFly [31] and Grey Wolf Optimization Algorithm (GWO) [32].

For other systems such as controlling the voltage regulator, Teaching Learning Based Optimization (TLBO) algorithm was used to optimize the parameters of PID controller [33]. Differential Evolution (DE) and its improved version (PSO-DE) [34] were used to optimize three liquid level tank systems.

The theory of No-Free-Lunch (NFL) [35] which states that “no one optimization algorithm can solve all engineering problems with the same efficiency”. Hence, upgraded versions of meta-inference based on embedding operators within algorithms or mixed meta-inference have been proposed to improve the algorithms for a particular engineering application. The nature of the optimization problem of the PID controller parameters is continuous because the search space has a large number of possible solutions which make the search fall into local minima besides its nonlinear behavior. Hence, a meta-heuristic algorithm containing efficient exploration and exploitation schemes is necessary to provide a better selection of PID controller parameters that achieved a better fit than those available in the literature.

Arithmetic Optimization Algorithm (AOA) [36] is a population-based algorithm that relies on the arithmetic operators of the search strategy. The main advantage of AOA is its effective exploration scheme which is defined as the ability of the search agents to visit most portions of the search space. However, the AOA exploitation scheme is poor resulting in a lack of microfitness and needs further enhancement. In this work, AOA has been improved by combining it with a heuristic algorithm which has an effective exploit strategy for tuning PID's parameters.

A lot of researches were done on AOA; a version of AOA released was used for multiobjective optimization [37]. AOA was used for multilevel threshold segmentation optimization problem of COVID-19 images [38]. Besides, AOA was used for truss optimization [39], optimal installation of distribution system [40], Fog computing [41], energy storage system [42], economic load dispatching [43], optimal power flow problem [44], brain computer interface [45], photovoltaic solar cell parameter extraction [46], optimal energy resource planning [47], intrusion detection system [48], and PEM fuel cell parameters estimation [49]. AOA was merged with Slime Mold Algorithm for global optimization [50]. The hybridization was tested on 23 mathematical benchmark functions and three classical engineering problems. AOA was merged with genetic algorithm for feature selection problem [51]. An improved version of AOA was released based on using high-density distribution function (beta distribution) to enhance the exploration scheme of AOA [52]. The enhanced version was tested on 30 mathematical benchmark function and engineering problems such as welded beam design, compression spring design and pressure vessel design. AOA was hybrid with Aquila optimizer for high-dimensional optimization problems [53]. A chaotic AOA was released for enhancing the speed convergence and avoiding local optima [54]. AOA was merged with differential evolution for truss structure optimization problem [55].

This paper presents a hybrid between AOA and Harris Hawk Optimization (HHO) [56] which has the benefits of efficient AOA exploration in addition to the efficient exploitation strategy of the hybrid algorithm. HHO algorithm is a population algorithm that inspired the attacking strategy of Harris Hawk for catching a prey [56]. The exploitation of search space by HHO is based on four schemes based on rabbit escape energy and rabbit escape chance. The four schemes diversify the movement patterns of hawksbill positions toward the best founding position (rabbit site), thus promoting intensification and head-avoidance in the local optima. HHO was used for optimizing many optimization problems such as feature selection [57], photovoltaic solar cell parameter extraction [58], color multilevel thresholding image segmentation [59], drug discovery [60], landslide susceptibility‏ [61], and passive suspension system [22].

HHO was used for enhancing the exploitation of many meta-heuristic algorithms such as Nelder–Mead simplex optimization algorithm [62], grasshopper optimization algorithm [63], Salp swarm optimization algorithm [64], and equilibrium optimization [65],

The main contributions of this work are listed as follows:

1. Enhanced AOA's exploitation by incorporating HHO for optimized PID controller design.
2. Avoidance of local AOA minima is enhanced based on the inclusion of disruption and mutation operators.
3. A DC motor and three liquid level tanks were used in pilot tests to test the performance of the developed AOA–HHO for the optimized design of the PID controller.

The rest of the paper is organized as follows: Sect. 2 describes the strategy of AOA and HHO, Sect. 3 describes the proposed hybrid algorithm (AOA–HHO), while Sect. 4 describes the procedure for estimating PID controller parameters based on AOA–HHO. Experimental results and discussion are presented in Sect. 5. Finally, the result of the proposed work is presented in Sect. 6.

## Preliminaries

In this section, the procedure of AOA [36] and HHO [56] is presented.

### AOA Algorithm

AOA uses arithmetic operators (addition, subtraction, multiplication, and division) to update solutions. To explore the search space, it depends on the multiplication and division operators, while the addition and subtraction operators are used for exploitation. The control parameter that balances diversification and intensification of search space is the accelerated Math Optimizer $({M}_{OA})$ which is described in Eq. (2).

2 $${M}_{OA}\left(t\right)=\frac{t}{T}$$

where $t$ is the number of current iteration and $T$ is the number of iterations.

Diversification of the search space based on division and multiplication factors is performed due to the high distribution of the generated values ​​which are represented according to Eq. (3) for a condition ($$rand>{M}_{OA})$$ where rand is a random generated number.

3 $${x_i}^{(t)} = \left\{ {\begin{array}{*{20}{c}}{{\rm{best}}\left( {{x^{(t)}}} \right){\rm{ }} \div (\left( {{M_{OP}} + \varepsilon } \right)\left( {\left( {{\rm{UB}} - {\rm{LB}}} \right)\mu  + {\rm{LB}}} \right))}&{{r_1} < 0.5}\\{{\rm{best}}\left( {{x^{(t)}}} \right) \times \left( {{M_{OP}}} \right)\left( {\left( {{\rm{UB}} - {\rm{LB}}} \right)\mu  + {\rm{LB}}} \right)}&{{\rm{else}}}\end{array}} \right\}$$

where $x$ is the solution, $i$ is the index of solution ($i$=1:*N*), best ($x$) is the best global solution, $\varepsilon$ and $\mu$ are constants, LB and UB are the lower and upper bound of the solutions, ${r}_{1}$ is a random generated number, Math Optimizer probability (${M}_{OP}$) is a scaling parameter that produces more exploration and is estimated according to Eq. (4) where α is a constant parameter.

4 $${M}_{OP} \left(t\right)=1- \frac{{t}^{1/\alpha }}{{T}^{1/\alpha }}$$

In terms of search space condensation, addition and subtraction operators are used due to the high density of solutions generated and implemented for a case ($$rand>{M}_{OA})$$. Equation 5 expresses the updating strategy during exploitation where $${r}_{2}$$ is a random generated number.

5 $${{x}_{i}}^{(t)}= \left\{\begin{array}{c}best \left({x}^{(t)}\right)-(\left({M}_{OP}\right) \left(\left(UB-LB\right) \mu +LB\right)) \quad {r}_{2}<0.5\\ best \left({x}^{(t)}\right)+ \left({M}_{OP}\right) \left(\left(UB-LB\right) \mu +LB\right) \quad else\end{array}\right\}$$

AOA’s procedure is listed in Algorithm 1.
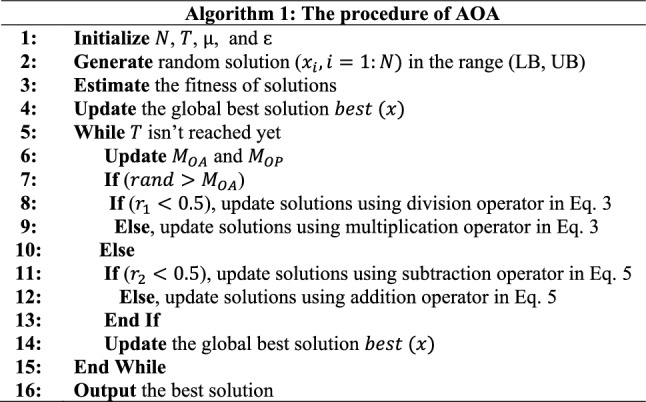


### HHO Algorithm

The HHO algorithm is a population-based heuristic that simulates the surprise-and-hunt mechanism of Harris Hooke. The prey is surprised by many hawks who cooperate to pounce on it, and according to the surrounding environment conditions and the escape method, the technique of chasing the hawk will be determined.

HHO's main feature is the hawks' cooperative way of attacking prey as more experienced hawks grab it. The attack mechanism and escape pattern of the prey are mathematically modeled where the search agents are represented by the hawks and the best solution is represented by the prey.

The HHO exploration stage is represented by the initial attack of the prey as it has a high energy which decreases during escape to a low energy level and can be attacked which represents the exploitation stage as shown in Eq. (6).

6 $${E}^{t}=2{E}_{0} (1-\frac{t}{T)}$$

where (E) represents the energy of the prey, (E_0_) is the initial energy of prey, and it has a value between (−1,1) which is assigned randomly, (t) is the current iteration number, and (T) is the total iterations number. For $(\left|E\right|>1)$, the exploration phase is executed, while for $(\left|E\right|\le 1)$ the exploitation phase is executed.

The exploration stage is carried out on two mechanisms: first, the falcons settle according to the positions of other falcons and the location of the prey. Second Mechanism Falcons can settle into a random position within the range of other falcons. Equation 7 simulates the exploration phase.

7 $${y}^{t+1}=\left\{\begin{array}{c}{{y}_{rand}}^{t}-{r}_{1} \left|{{y}_{rand}}^{t}-2{r}_{2}{y}^{t} \right| \quad c\ge 0.5 \\ {{(y}_{rabbit}}^{t}- {{y}_{av}}^{t})- {r}_{3} (LB+{r}_{4}(UB-LB)) \quad c<0.5\end{array}\right\}$$

where ${y}^{t+1}$ is the solutions in the next iteration of search agents, ${{y}_{rand}}^{t}$ is a hawk of the search agents is selected randomly, ${y}^{t}$ is the solutions of the search agents at iteration (t), ${{y}_{rabbit}}^{t}$ is the global solution among the search agents, and ${{y}_{av}}^{t}$ is the average of all search agents’ solutions at iteration (t). r_1_, r_2_, r_3_ and r_4_ and are random generator numbers within the range (0,1).

During the exploitation phase $\left(\left|E\right|\le 1\right),$ the hawks can attack hardly $(\left|E\right|<0.5)$ or softly$(\left|E\right|\ge 0.5)$; in addition, there is a probability for escapping for the rabbit (r) where $(r<0.5)$ the rabbit escapes successfully and for $(r\ge 0.5)$ it is catched. The various chasing style according to the energy (E) and the probability of escaping ( r) are as follows:

#### A. Un-successful escaping $({\varvec{r}}\ge 0.5)$ and Soft besieges $(\left|{\varvec{E}}\right|\ge 0.5)$

Hawks surround the rabbit while it tries to escape and is tired and then pounced by the hawks. This process is simulated as in Eq. (8).

8 $${y}^{t+1}={{(y}_{\mathrm{rabbit}}}^{t}- {y}^{t})-E \left|J {{y}_{\mathrm{rabbit}}}^{t}- {y}^{t}\right|$$

where (J) simulates the rabbit's jump power randomly through escaping and J = 2(1-r_5_), r_5_ is a parameter its value is selected randomly within the range [0,1].

#### B. Successful escaping $\left({\varvec{r}}<0.5\right)$ and Soft besieges $\left(\left|{\varvec{E}}\right|\ge 0.5\right)$

The rabbit (the prey) escapes in a zigzag pattern simulated using mega-flying. The hawks search for the best direction of attack to catch the hare so that the hawks decide the next attack according to Eq. (9).

9 $$X={{y}_{\mathrm{rabbit}}}^{t}- E \left|J {{y}_{\mathrm{rabbit}}}^{t}- {y}^{t}\right| $$

Levy's flight function is used to simulate the random and irregular attacks of hawks to capture prey according to Eq. (10).

10 $$Z=X+s \times LF( )$$

where s is a random value generated within the range (0,1) and LF() is the levy flight function represented according to Eq. (11).

11 $$LF\left( {} \right) = 0.01 \times \frac{{u \times \sigma }}{{{{\left| v \right|}^{\frac{1}{\beta }}}}},\sigma  = {\left( {\frac{{\Gamma \left( {1 + \beta } \right) \times {\rm{sin}}(\frac{{\pi \beta }}{2})}}{{\Gamma \left( {\frac{{\left( {1 + \beta } \right)}}{2}} \right) \times \beta  \times {2^{(\frac{{\beta  - 1}}{2})}}}}} \right)^{\frac{1}{\beta }}}$$

where β is a set constant as a value (1.5) and u and v are randomly generated values ​​within the range (0,1). The position of the falcon is estimated according to Eq. (12).

12 $${y}^{t}= \left\{\begin{array}{c}X  \quad if F\left({y}^{t}\right)>F(X)\\ Z  \quad if F\left({y}^{t}\right)>F(Z)\end{array}\right\}$$

where *Z* and *X* are estimated according to Eq. (10) and Eq. (9) in order.

#### C. Un-successful escaping $\left({\varvec{r}}\ge 0.5\right)$ and Hard besieges $\left(\left|{\varvec{E}}\right|<0.5\right)$

The prey is ejected from the air and has a low energy to lunge, so the hawk veers strongly toward the hare to carry out Sally's attack. The update of the current position of the hawk toward the hare is simulated according to Eq. (13).

13 $${y}^{t+1}={{y}_{\mathrm{rabbit}}}^{t}-E \left| {{y}_{\mathrm{rabbit}}}^{t}- {y}^{t}\right|$$

#### D. Successful escaping $\left({\varvec{r}}<0.5\right)$ and Hard besieges $\left(\left|{\varvec{E}}\right|<0.5\right)$

The hawk glides toward the hare aggressively in order to deflate, but it attempts to limit prey by a small distance to the hawk's average positions. Besides, levy flight is used to simulate the zigzag way of prey and the rare movements of falcons. Equations (14) and (15) update the situation, while Eq. (16) determines the final position.

14 $$X={{y}_{rabbit}}^{t}- E \left|J {{y}_{rabbit}}^{t}- {{y}_{av}}^{t}\right|$$

15 $$Z=X+s \times LF( )$$

16 $${y}^{t}= \left\{\begin{array}{c}X \quad if F\left({y}^{t}\right)>F(X)\\ Z \quad  if F\left({y}^{t}\right)>F(Z)\end{array}\right\}$$

Algorithm (2) represents the steps of HHO algorithm.
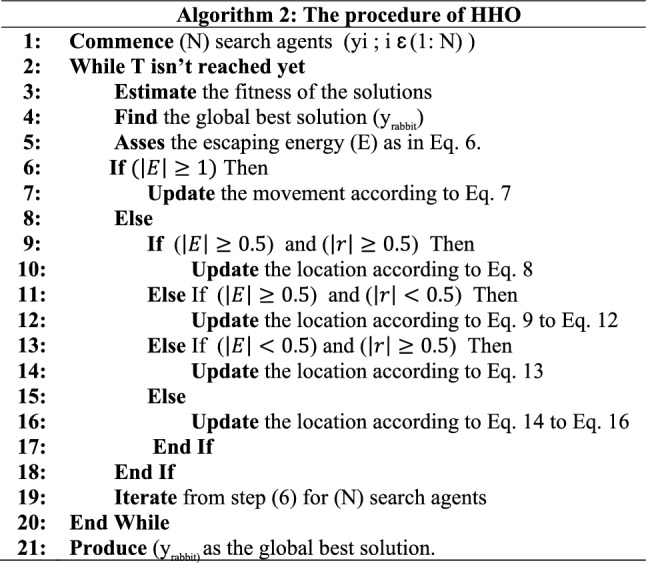


## The Proposed Hybrid Algorithm (AOA–HHO)

The main advantage of AOA is efficient exploration due to division and multiplication operators due to their strong ability to generate values ​​with high distribution. However, it produced poor performance for PID controller's parameters estimation because it is trapped in local minima and has poor exploitability. Hence, this is the impetus to enhance avoidance of local minima by using the disruption operator and the mutation operator. Its poor exploit is improved by incorporating the exploitation scheme of the HHO algorithm (Heidari et al., 2019) where its main advantage is the different efficient exploitation mechanisms that balance the search focus on narrow areas and the avoidance of trapping in the local minima. The disruption factor and mutation operator are described in subsections 3.1 and 3.2, in order. In subsection 3.3, the proposed AOA–HHO hybrid procedure is described.

### Disruption Operator

The disruption operator inspired by an astrophysical theory posits that “when a group of gravitationally bound particles (with a total mass m) is very close to a massive object (with a mass of M), the group becomes torn apart. Similar to this, when a solid body, held together by gravitational forces, approaches a much larger body” [66]. The disruption operator is used to enhance the diversity of the search space where the disruption operator is mathematically modeled as in Eq. (17) [67].

17 $${D}_{OP}= \left\{\begin{array}{c}{D}_{i,j} \times  U\left(-\mathrm{2,2}\right) if {D}_{i ,\mathrm{ best}}\ge 1\\ 1+ {D}_{i ,\mathrm{ best}} \times  U\left(-\frac{{10}^{-4}}{2},\frac{{10}^{-4}}{2}\right) Otherwise\end{array}\right.$$

${D}_{OP}$ represents the disruption operator, and ${D}_{i , j}$ is the distance between the search agent (i) and nearest neighborhood search agent (j). ${D}_{i , best}$ is the distance between the search agent (i) and the best solution (best). $\mathrm{U }(x,y)$ is a number generated randomly withing the range$(x,y)$. For updating the solutions, the disruption operator is used to enhance its diversity as in Eq. (18).

18 $$X= \left\{\begin{array}{c}{X}^{\mathrm{past}} \times  {D}_{OP} if \alpha >0.5\\ {X}^{\mathrm{past}} Otherwise\end{array}\right.$$

where *X*^*past*^ represents current value of the solution to be updated, X is the value after updating the solution, and α is a random number generated within the range (0,1). The disable factor has successfully improved many meta-algorithms in many applications such as feature selection [68, 69], optimal flow problem [70], image thresholding [71] and photovoltaic solar cell design [72].

### Mutation Operator

The mutation factor is used to enhance the diversity of the search space and to avoid falling into local lower bounds where two well-known factors are the Cauchy mutation (CM) and the Gaussian mutation (GM). The mutation factor has been used to enhance the diversity of several heuristic algorithms in the literature that has motivated their use [73–78].

CM operator has better ability of efficient search than GM operator according to previous research [73, 77, 79, 80]. Since the CM operator has a wider distribution of searching in the horizontal direction than the vertical direction in contrast to the GM operator, so it is the main motive for the use of the CM operator.

The CM operator’s density function is represented as follows as shown in Eq. (19):

19 $${f_{\left( {0,g} \right)}} \left( \gamma  \right) = \frac{g}{\pi (g + \gamma ^{2})}, \gamma  = {\rm{tan}}(\pi ({\rm{rand}} - 0.5))$$

where g is the constant parameter with value (1) [77] and rand is a random number within the range (0,1).

### The Proposed AOA–HHO Procedure

**Fig. 3:**
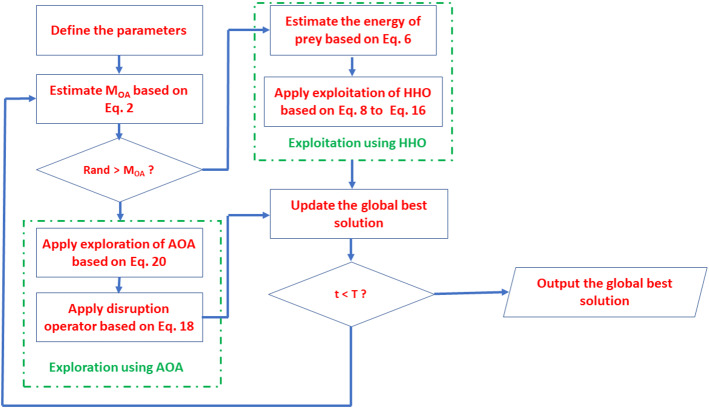
The procedure for the proposed hybrid AOA–HHO algorithm

The enhancement of AOA was performed by embedding the CM operator in the exploration updating mechanism of AOA as in Eq. (20).

20 $${x_i}^{(t)} = \left\{ {\begin{array}{*{20}{c}}{{\rm{best}}\left( {{x^{(t)}}} \right){\rm{ }} \div \left( {{M_{OP}} + \varepsilon } \right) \times {\rm{CM}} \times \left( {\left( {{\rm{UB}} - {\rm{LB}}} \right)\mu  + {\rm{LB}}} \right)}&{{r_1} < 0.5}\\{{\rm{best}}\left( {{x^{(t)}}} \right) \times \left( {{M_{OP}}} \right) \times {\rm{CM}} \times \left( {\left( {{\rm{UB}} - {\rm{LB}}} \right)\mu  + {\rm{LB}}} \right)}&{{\rm{else}}}\end{array}} \right\}$$

where CM is the mutation factor and was estimated based on Eq. (19). Then the inactivation factor was applied by implementing Eq. (18). During the exploit phase, the AOA exploit mechanism (Eq. 4) was replaced by the update mechanism exploited the HHO algorithm (Eq. 8 to Eq. 16). The MOA operator that is estimated according to Eq. (2) balance between exploration and exploitation. The AOA–HHO hybrid procedure is described in Algorithm (3). The flowchart describing the proposed AOA–HHO procedure is shown in Fig. 3.

The main advantages of the proposed AOA–HHO algorithm are inferred in the following:

1. Promoting trapping avoidance in the local minima based on the use of CM operator.
2. Enhancing the diversification of the search space based on the disruption factor.
3. Enhancing the exploitation system based on the mechanism of exploiting of the HHO algorithm.

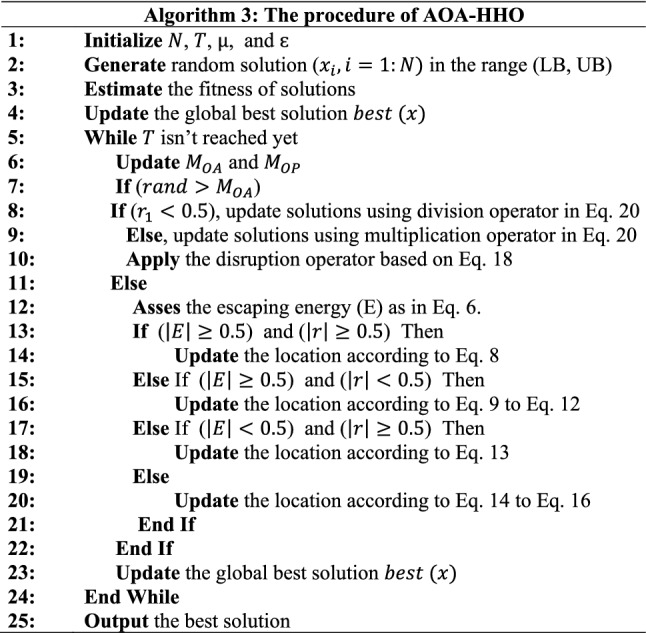


## PID’s Parameter Estimation Based on AOA–HHO

**Fig. 4:**
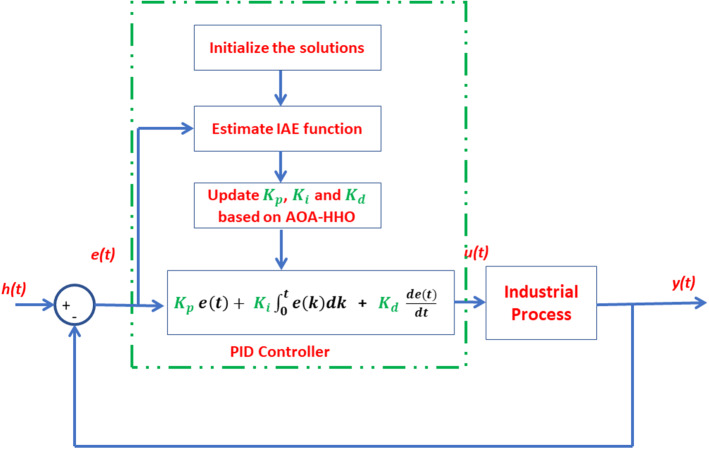
Estimation of PID parameters based on the proposed AOA–HHO algorithm


The integral of the absolute error (IAE) function represented in Eq. (1) was used as a fitness function for PID controller's parameters estimation based on the proposed AOA–HHO algorithm shown in Fig. 4. Each AOA–HHO search factor has a vector of three values (${K}_{p}$, ${K}_{i}$ and ${K}_{d}$), and each solution is initialized with a random value in the lower and upper bounds. The IAE function is estimated based on the sign of e(t) for each research agent to evaluate solutions. The best solution${({K}_{p}}^{g}, {{K}_{i}}^{g}, {{K}_{d}}^{g}$) is determined based on the search agents solution that achieved minimum relevance. The variable MOA controls the implementation of the exploration phase using the AOA or the exploit phase of the HHO algorithm. After completing the execution of iterations, the best solution${({K}_{p}}^{g}, {{K}_{i}}^{g}, {{K}_{d}}^{g}$) has been founded. Algorithm (4) describes the procedure for estimating the parameters of the PID controller using the proposed AOA–HHO algorithm.

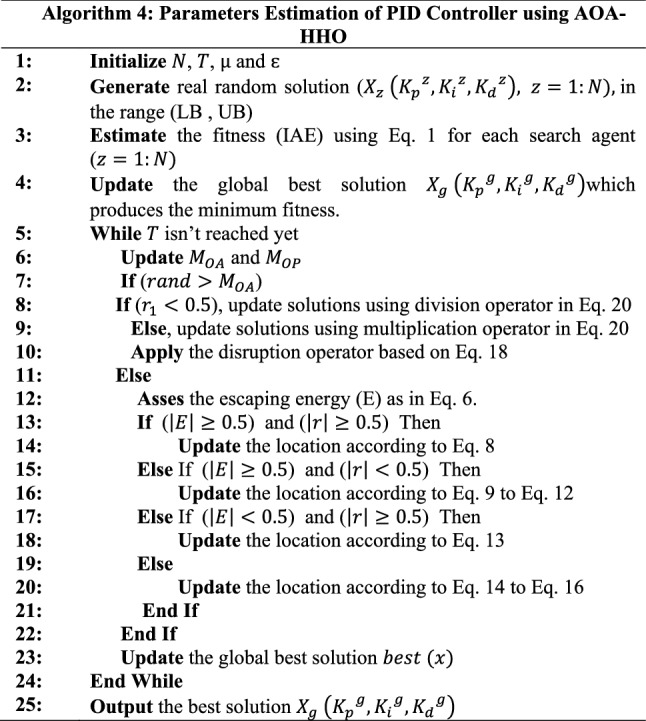


## The Experimental Results and Discussion

Experimental tests were carried out on two systems: the first was DC motor speed control, and it was common in many related studies [18, 27–29, 32, 81]. The second system was controlling the liquid level for three consecutive tank systems [34]. The experimental results were compared with relevant studies such as PSO [6], SCA [5], IWO [29], GWO [32], ASO, PSO-DE [34] and OBL-HGS [27] and Covariance Matrix Adaptation Evolution Strategy (CMA-ES) [82].

**Fig. 5:**
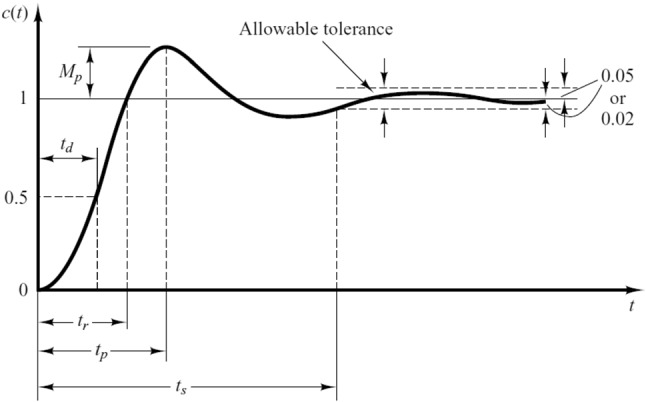
Time domain specification of controlled process response

The step response characteristics of controlled process response in time domain are delay time, rise time, peak time, settling time and overshooting as shown in Fig. 5 which are defined as follows [1]:

1. Delay time (t_d_): it is the time required for the response to achieve half of the final value for the first time.
2. Peak time (t_p_): it is the time required for the response to reach the first peak of the overshoot.
3. Rise time (t_r_): it is the required time for the response to rise from 10% to 90 of its final value.
4. Settling time (t_s_): it is the time required for the response curve to reach and stay within a range about the final value of size specified by absolute percentage of the final value (usually 2% or 5%).
5. Overshooting (M_p_): it is the maximum peak value of the response curve measured from unity.

The fitness function that was used to evaluate solutions based on IAE according to Eq. (1). The measurement criteria that were used in the comparisons are as follows:

1. Integral absolute of difference error between actual and desired responses (IAE)
2. The step response characteristics as settling time, rise time and overshooting.
3. The frequency response of the systems.

### Speed Regulator of DC Motor System

The speed regulation of electrical DC motor [27] was controlled by a PID controller where heuristic algorithms were used to select the best parameters that produce the optimal response. Parameters setting of PSO, CMA-ES, AOA and the proposed AOA–HHO algorithm are listed in Table 1 which were estimated experimentally to get the best results. The results of other algorithms (SCA, IWO, GWO, ASO, and OBL-HG) were obtained from their origin manuscript.

**Table 1:** The parameters setting of various algorithm for DC motor

	The parameter	Value
All algorithms	The population (N)	100
	Iteration Number (T)	20
	Independent run number	20
	Lower bound of $({\mathrm{K}}_{\mathrm{p}}$, ${\mathrm{K}}_{\mathrm{i}}$ and ${\mathrm{K}}_{\mathrm{d}})$	[0.001,0.001,0.001]
	Upper bound of $({\mathrm{K}}_{\mathrm{p}}$, ${\mathrm{K}}_{\mathrm{i}}$ and ${\mathrm{K}}_{\mathrm{d}})$	[20, 20, 20]
PSO	C_1_	0.5
	C_2_	0.5
	w	0.1
CMA-ES	${C}_{o^\prime}$	0.2
	${C}_{\mu }$	0.2
	${\mu }_{\omega }$	0.5
AOA–HHO, AOA	$\mu$	0.5
	$\varepsilon$	2

The values of the parameters of DC motor which was used as a case study are listed in Table 2 [32]. R_a_ represents armature resistance, L_a_ represents inductance of armature winding, J represents the equivalent moment of inertia of motor and load referred to motor shaft, D is the equivalent friction coefficient of motor and load referred to motor shaft, K represents Motor torque constant, and K_b_ represents back EMF constant.

**Table 2:** Parameters of DC motor [32]

Parameter	Value
R_a_	0.4 Ω
L_a_	2.7 H
J	0.0004 kg. m^2^
D	0.0022 N.m.sec / rad
K	15 e − 03 kg. m / A
K_b_	0.05 V.s

The transfer function of DC motor closed-loop speed control systems is expressed in Eq. (16).

21 $${G}_{1}\left(S\right)= \frac{15}{{1.08 s}^{2}+6.1 s+1.63}$$

Table 3 presents the best PID controller parameter values for optimizing DC motor speed regulation using AOA–HHO versus standard AOA, and other related algorithms were used in the comparative study. AOA–HHO optimizes single objective which is IAE where the solution is the best parameters of PID controller that achieve the minimum IAE. Other specifications such as set time, rise time and overshoot were measured according to the estimated parameters for AOA–HHO and other algorithms in the comparative.

**Table 3:** Step response and IAE specification for various heuristic algorithms

Method	Kp	Ki	Kd	Set Time (Sec)	Rise Time (Sec)	Over-shoot %	IAE
PSO	1.5234	0.4372	0.0481	0.3549	1.8016	24	12.36
SCA [83]	4.5012	0.5260	0.5302	0.2037	0.4900	2.36	13.63
IWO [29]	1.5782	1.3801	0.0159	0.4190	1.2533	6.7	18.55
GWO [32]	6.898	0.5626	0.9293	0.1388	0.2053	1.5	10.99
ASO [84]	11.943	2.0521	2.4358	0.0692	0.1535	0	22.27
CMA-ES	17.3347	10.9710	0.2140	0.8170	0.0800	44.46	14.73
OBL-HG [27]	16.9327	0.9508	2.8512	0.0546	0.0949	0	21.58
AOA AOA-HHO	17.057	4.8488	0.2917	0.7135	0.0821	37.75	14.6156
	14.435	0.1636	1.7620	0.2508	0.0743	2.83	9.0465

As shown in Table 3, AOA–HHO has the superiority over other algorithms for finding the minimum IAE. It enhances the IAE of AOA from 14.6156 to 9.0465 which proves the enhancement of AOA–HHO using disruption and mutation operator for enhancing exploration and HHO algorithm for enhancing exploitation. PSO and GWO found the most nearest value of IAE to that founded by AOA–HHO, but that of other algorithms is far. AOA–HHO provides IAE better than that of the hybrid techniques such as CMA-ES and OBL-HG which prove the powerful of hybrid technique between AOA and HHO.

**Fig. 6:**
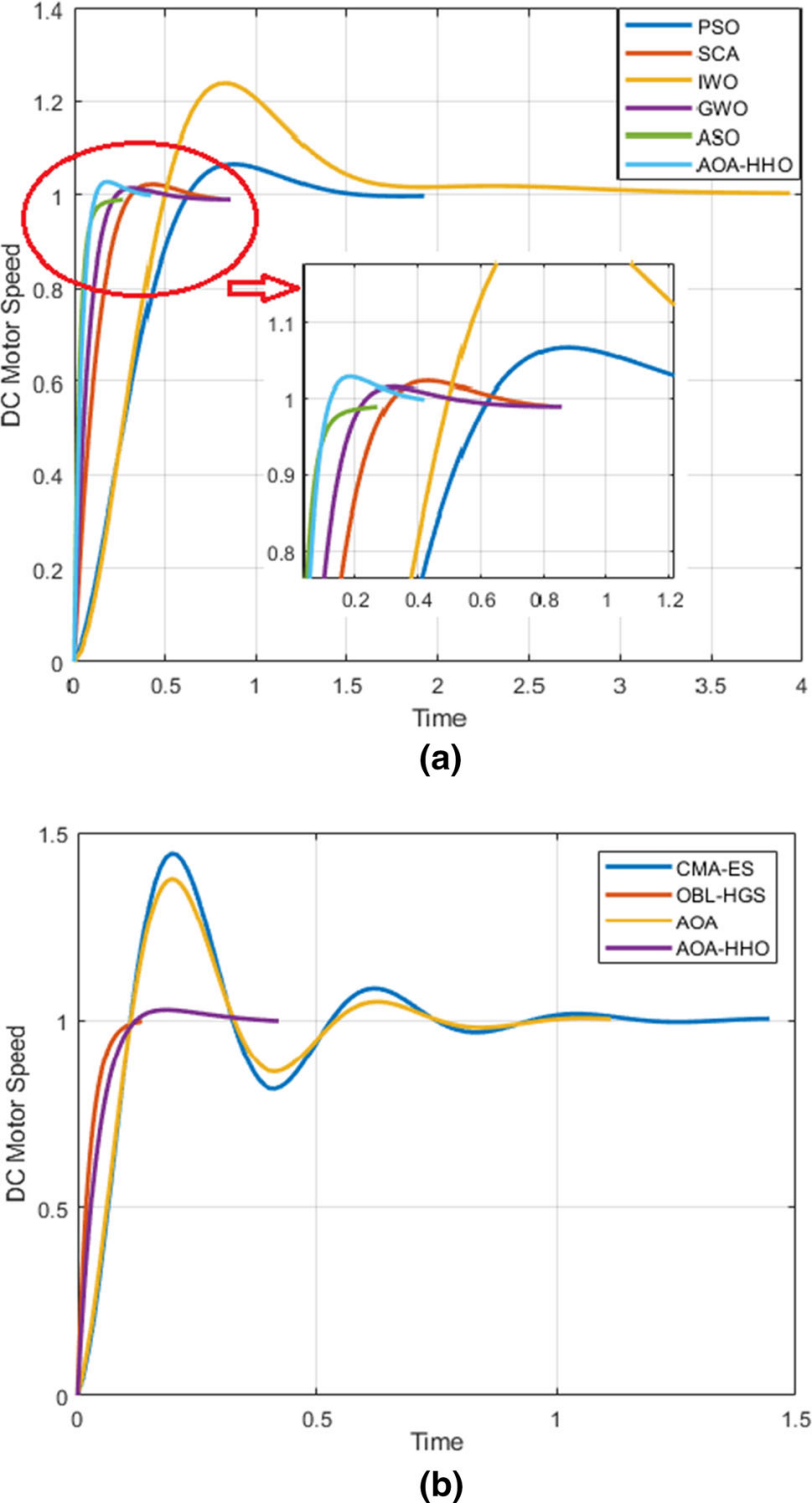
DC motor response versus time in seconds

For overshoot measurement, AOA–HHO provides overshoot very smaller than that of AOA but not the optimum due to that of GWO and SCA is smaller as shown in Fig. 6. The overshoot of parameters estimated by ASO and OBL-HG is 0 which implies that the system response in this case is over-damping which is the best response shape, but IAE of ASO and OBL-HG is larger than that of AOA–HHO. The reason as mentioned before the single objective was optimize IAE not overshoot.

For rise time, AOA–HHO produces the smallest rise time according to results of Table 3, while PSO provides the largest one. That is guaranteed from Fig. 6. For set time, AOA–HHO provides set time smaller than that of AOA, PSO, SCA, IWO and CMA-EA. However, set time of AOA–HHO is not the smallest, but it is reasonable because it implies the time needed for the response to be set around the set point with a percentage 2% or 5%.

This results implies that AOA–HHO has the superiority over than AOA and other algorithms for finding the smallest IAE and reasonable overshoot, set time and rise time.

**Fig. 7:**
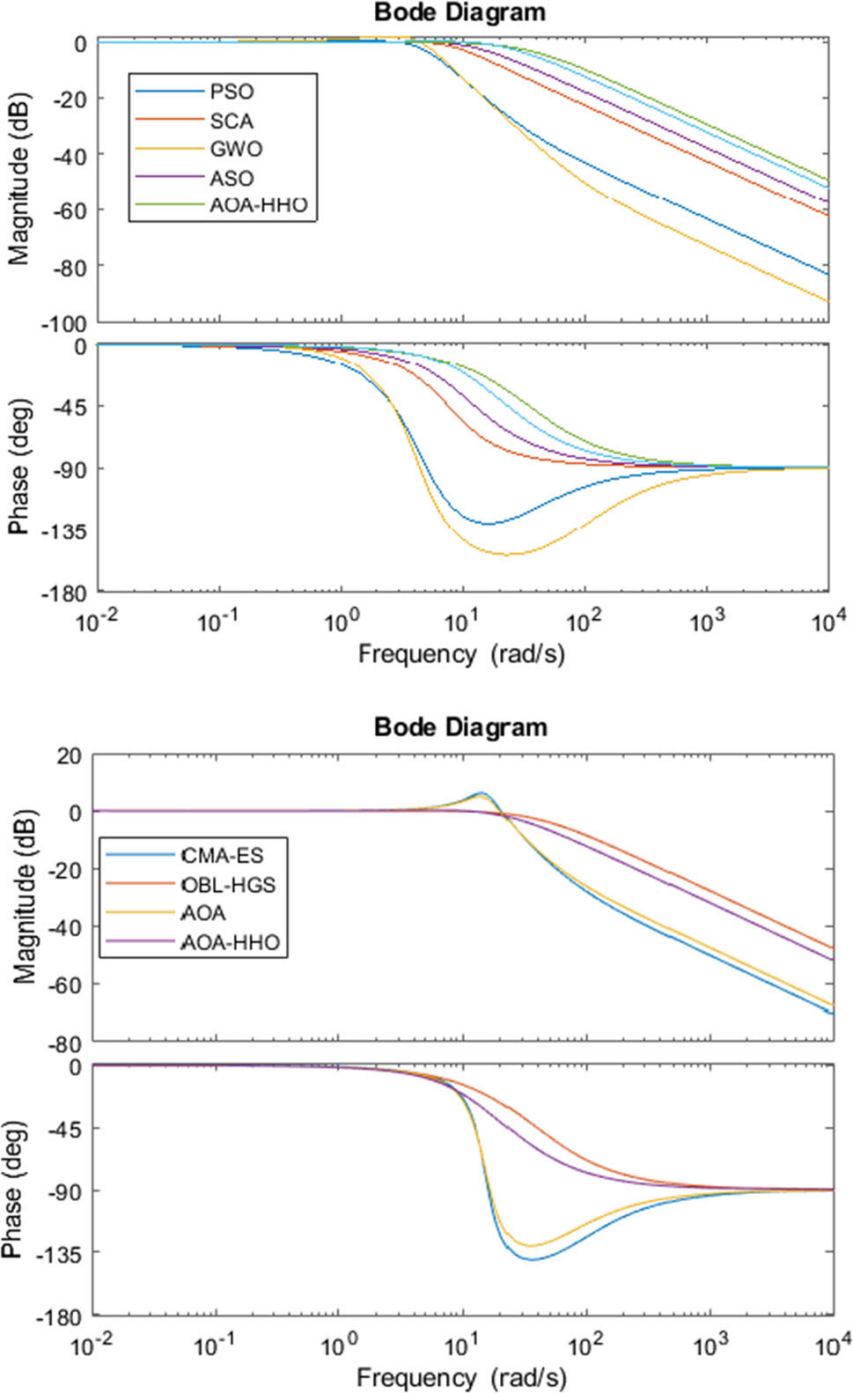
Bode plots for DC motor based on PID controller

Figure 7 shows the Bode diagrams for regulating a DC motor using a PID controller where its parameters are calculated using the proposed AOA–HHO algorithm and related study algorithms. As shown, AOA–HHO has a wider bandwidth better than that of algorithms used in the comparison except OBL-HGS. This guarantees that AOA–HHO has smaller rise time than other algorithms except OBL-HGS as shown in Table 3 and Fig. 6.

In addition, the magnitude margin of AOA–HHO has not any gain, while that of AOA and CMA-ES has a gain which implies that it produces larger overshoot which is guaranteed as shown in Fig. 6.

### Liquid Level Tank

**Fig. 8:**
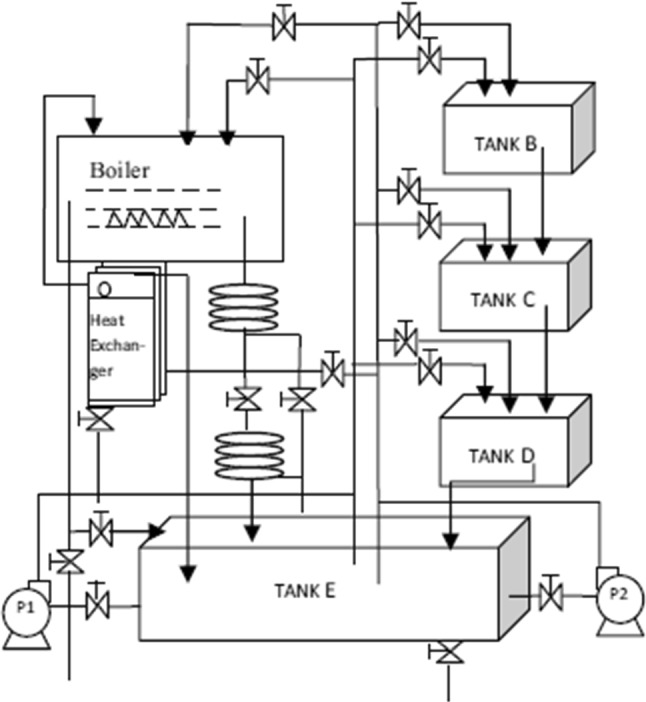
Three cascaded tanks liquid level systems [34]

Three cascaded liquid level tank system was used to test the performance of improved DE with PSO [34] for estimating the parameters of PID controller. As shown in Fig. 8, three tanks B, C and D are cascaded, while tank E is the main tank. Equation 17 expressed the transfer function of the liquid level tank systems [34]. Table 4 presents the best estimated parameters of the PID controller using AOA–HHO and related algorithms that were used in the experimental tests. Besides, the step response characteristics have been added in the table as well as the trap value (IAE).

22 $$ {G}_{2}\left(S\right)= \left( \frac{1}{4s + 0.2} \right)^{3} = \frac{1}{64s^{3} + 9.6s^{2} + 0.48s + 0.008}$$

**Table 4:** The parameters setting of various algorithms for liquid level control

	The parameter	Value
All algorithms	The population (N)	100
	Iteration Number (T)	20
	Independent run number	20
	Lower bound of $({\mathrm{K}}_{\mathrm{p}}$, ${\mathrm{K}}_{\mathrm{i}}$ and ${\mathrm{K}}_{\mathrm{d}})$	[0.001,0.001,0.001]
	Upper bound of $({\mathrm{K}}_{\mathrm{p}}$, ${\mathrm{K}}_{\mathrm{i}}$ and ${\mathrm{K}}_{\mathrm{d}})$	[20, 20, 20]
PSO	C_1_	0.5
	C_2_	0.5
	w	0.1
SCA	a	3
GWO	$\overline{{a }_{0}}$	2
ASO	α, β	30
	δ	4
CMA-ES	${C}_{o^\prime}$	0.2
	${C}_{\mu }$	0.2
	${\mu }_{\omega }$	0.5
AOA–HHO, AOA	$\mu$	0.5
	$\varepsilon$	2

Parameters setting of PSO, SCA, GWO, ASO, CMA-ES, AOA and the proposed AOA–HHO algorithm are listed in Table 4 which were estimated experimentally to get the best results. The results of PSO-DE were obtained from their origin manuscript.

As shown in Table 5, AOA–HHO has the minimum IAE which is better than that of AOA which has the largest IAE between the comparative algorithms. PSO-DE and GWO produce the most nearest IAE to that of AOA–HHO which is 9.13 and 10.76 in order.

**Table 5:** Step response specification and IAE using meta-heuristic algorithms

Method	K_p_	K_i_	K_d_	Set time (sec)	Rise time (Sec)	Over-shoot %	IAE
PSO	0.6060	0.0024	14.4250	80.68	2.4078	67.86	18.61
SCA	0.3039	0.1154	6.7231	207.18	3.5526	4.428	15.66
GWO	0.2928	0.0396	4.719	89.51	4.2374	73.22	10.76
ASO	0.1642	0.0048	12.922	70.1	2.6376	57.26	14.47
CMA-ES	0.051	0.0013	0.3914	238.58	15.0019	50.08	14.27
PSO-DE [34]	0.0419	0.0009	1.000	64.21	12.7790	12.45	09.13
AOA	0.407	0.1184	12.066	2.649	82.756	71.63	16.865
AOA–HHO	0.040	0.0005	0.4269	160.363	17.7783	20.2	8.293

For overshoot, AOA–HHO cannot produce the minimum value, but it is better than that of AOA by difference 50%. SCA produces the minimum overshoot, while it produces high value of IAE. GWO produces the largest overshoot, while it produces reasonable value of IAE. The reason is that the single objective is minimizing IAE not the specification of the response, which can be enhanced in the future work by handling the problem as multi-objectives.

For rise time, AOA–HHO produces reasonable rise time not the minimum but better than that of AOA by difference 65 s. PSO produces the minimum rise time, while AOA produces the largest value of rise time.

For set time, AOA–HHO produced 160.363 s of set time which is larger than that of AOA which has value of 2.65 s. CMA-ES produced the largest value of set time, while AOA produced the minimum value of set time.

**Fig. 9:**
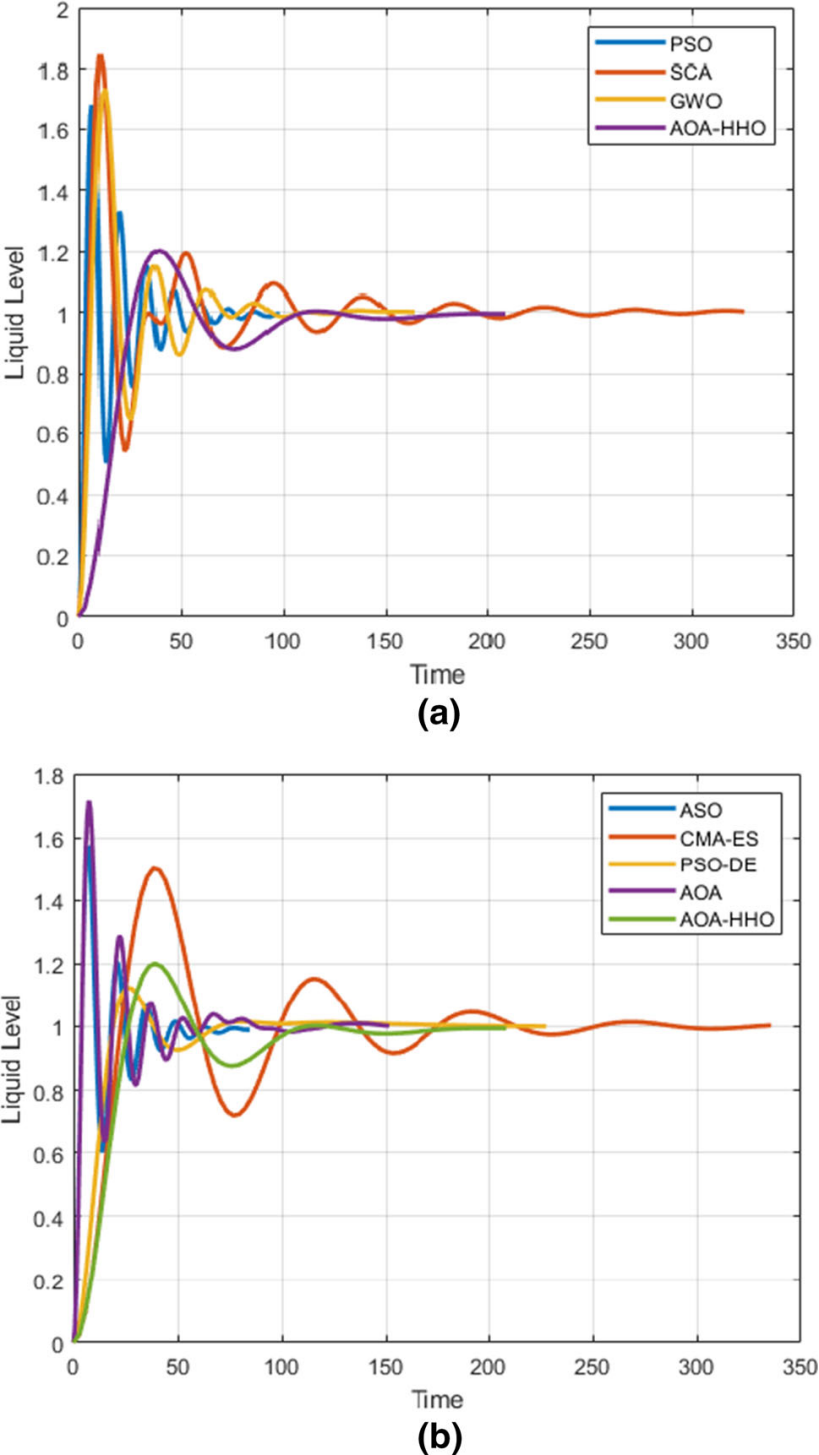
Liquid level response versus time in seconds

**Fig. 10:**
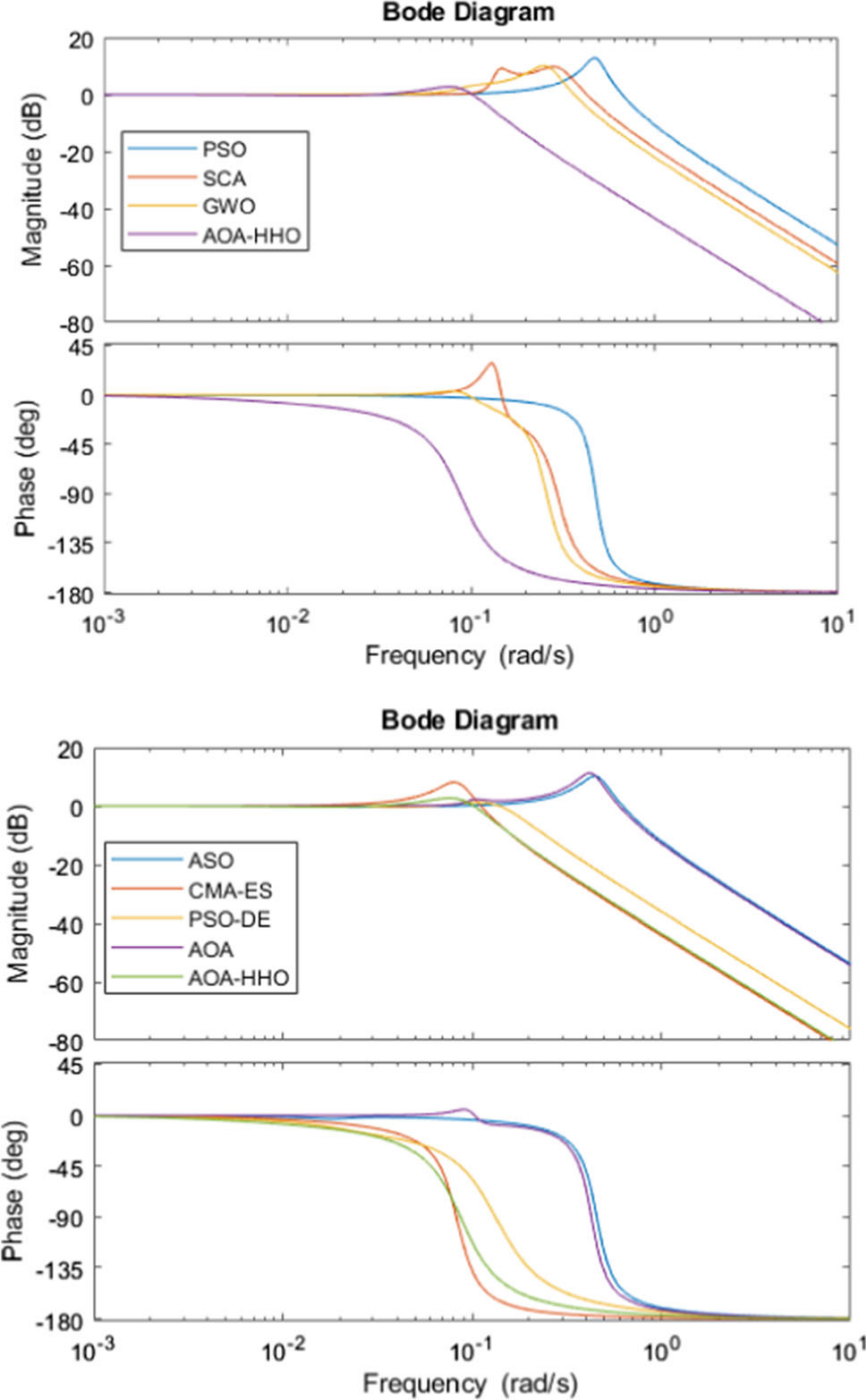
Bode plots for liquid level tank system based on PID controller

Figure 10 shows Bode diagrams for three liquid level tank systems using a PID controller where its parameters are calculated using the proposed AOA–HHO algorithm and related study algorithms. As shown in the figure, the proposed AOA–HHO algorithm has narrower bandwidth than other algorithms; hence, it produces larger rise time as shown in Table 4 and Fig. 9. In addition, according to the magnitude margin of AOA–HHO it has small gain in comparison with that of other algorithms, which implies it has smaller overshoot as shown in Table 4 and Fig. 9.

As shown from the results of the two case studies, AOA–HHO has the superiority over than AOA and other algorithms. The reason is that tuning the parameters of PID controller is a continuous optimization problem which has huge number of allowable values in determined search space. Hence, the balance between exploration and exploitation is an important for achieving the optimal values. In AOA–HHO, Cauchy mutation operator enhances the avoidance of trapping in local optima, while disruption operator enhances the exploration capability of AOA. These two operators enhance the diversification of the AOA–HHO. Regarding the exploitation, HHO was integrated which has an efficient exploitation schemes (Fig. 10).

## Conclusion

In this work, an enhanced version of AOA is presented to improve the estimation of PID controller parameters. The improvements were made by incorporating the efficient exploitation mechanism of the HHO algorithm instead of exploiting the AOA. In addition, trapping avoidance in the local minima of the proposed AOA–HHO algorithm was enhanced by including disruption and mutation factors which enhance the exploration capability. The AOA–HHO has been tested to select the best parameters of the PID controller for controlling two engineering applications that are DC motor regulation and three cascading liquid level tank systems. The single objective function was an integral part of the absolute error (IAE) function.

AOA–HHO has outperformed AOA in terms of IAE and response specification such as overshoot, rise time and set time in controlling dc motor, while for controlling level of three cascaded liquid tanks only set time of AOA is better that that of AOA–HHO. The frequency response of AOA–HHO was measured which implies it produces reasonable bandwidth and gain magnitude margin better than that of AOA and other comparative algorithms. From the experimental study, AOA–HHO has the superiority over AOA and other comparative study for estimating efficient parameters of PID controlling which leads to efficient IAE.

## Abbreviations

ZN: Ziegler–Nichol
SCA: Sine–cosine optimization algorithm
PSO: Particle swarm optimization
IMO: Ions motion optimization
MFO: Moth-flame optimization
IAE: Integral absolute error
dPSO: Dynamic PSO
IWOA: Improved whale optimization algorithm
OBL-HGS: Opposition Henry gas solubility
IWO: Invasive weed optimization
FPA: Flower pollination algorithm
GWO: Grey Wolf optimization
TLBO: Teaching learning based optimization
DE: Differential evolution
NFL: No-free-lunch
HHO: Harris Hawk optimization
CM: Cauchy mutation
GM: Gaussian mutation
